# Analysis of Anthocyanins Extracted from the Acai Fruit (*Euterpe oleracea*): A Potential Novel Vital Dye for Chromovitrectomy

**DOI:** 10.1155/2018/6830835

**Published:** 2018-07-19

**Authors:** Cristiane S. Peris, Rafael R. Caiado, Acácio Alves Souza Lima-Filho, Eduardo B. Rodrigues, Michel Eid Farah, Mariana Batista Gonçalves, Bruno de Queiroz Alves, Joao Guilherme Palma Urushima, Raul Ragazzi, Mauricio Maia

**Affiliations:** ^1^Department of Ophthalmology, Federal University of São Paulo, Botucatu Street, 816–Vila Clementino, 04023-062 São Paulo, SP, Brazil; ^2^Ophthalmos S/A, Brigadeiro Luís Antônio Avenue, 4830–Jardim Paulista, 01401-002 São Paulo, SP, Brazil; ^3^Brazilian Institute of Fight Against Blindness, Otto Ribeiro Avenue, 901–Jardim Paulista, 19814-470 Assis, SP, Brazil

## Abstract

**Purpose:**

To classify and quantify anthocyanins in a vital dye extracted from the acai fruit (*Euterpe oleracea*), adjust pH and osmolarity, and perform lyophilization to develop a new chromovitrectomy dye.

**Methods:**

Three dye concentrations 10%, 25%, and 35% (equivalent to 100, 250, and 350 mg of lyophilized acai fruit pulp extract samples) were evaluated when diluted in 1 ml of phosphate-buffered solution (pH 7 and 300 mOsm). The dye was analyzed by mass spectrometry and high-performance liquid chromatography (HPLC) to identify and quantify anthocyanins molecules.

**Results:**

The pH and osmolarity correction and lyophilization were performed without damaging the anthocyanin molecular structure. Mass spectrometry confirmed the presence of five anthocyanins in the three concentrations of the dye. Cyanidin-3-*O*-glucoside was the major anthocyanin found. HPLC showed that the concentration of anthocyanin was similar, independent of the dye concentration tested.

**Conclusions:**

Lyophilization and the correction of pH and osmolarity (7.00 and 300 mOsm, resp.) were performed successfully. Five anthocyanins are present in the dye from the acai fruit. The major anthocyanin is cyanidin-3-*O*-glucoside. Independent of the dye concentration tested, the anthocyanin concentration was similar. Standardized chemical characteristics of this new dye may allow its use during chromovitrectomy in humans.

## 1. Introduction

The acai fruit (*Euterpe oleracea*) from the palm tree is native to the Amazon forest in Brazil. In addition to being one of the main trees that produce palm hearts, the acai palm also produces a fruit with high nutritional properties and economic value for the food and cosmetic industries. The acai fruit has been described as caloric and rich in proteins, polyunsaturated fatty acids, and sugars (Table 1) [1].

**Figure 1:**
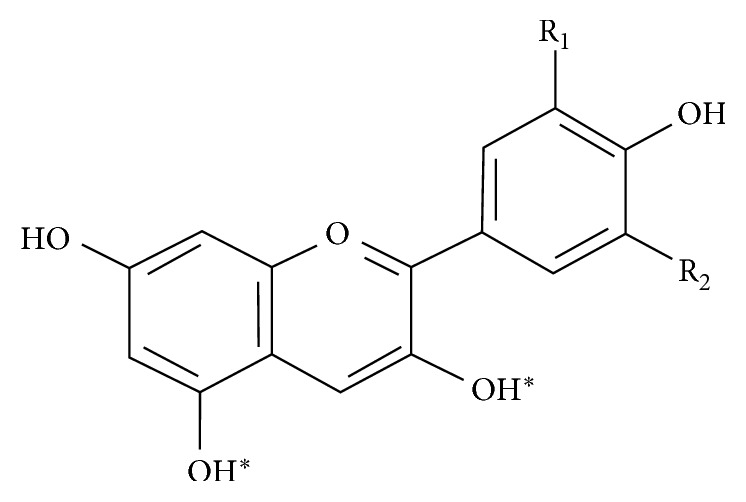
Basic molecular structure of the anthocyanins.

Carotenoids and anthocyanins are among the main components of the acai fruit [2–4]. Anthocyanins, derived from the Greek words anthos (flower) and kianos (blue), are part of a large group of organic components known as flavonoids that are represented by the basic chemical structure C6-C3-C6 (Figure 1). The phenolic structure of an anthocyanin provides an antioxidant effect due to electron donation or transference of hydrogen atoms.

It has been suggested that photosensitizing dyes used in chromovitrectomy could enhance phototoxicity by increasing levels of free radicals, creating a photoproduct that could be harmful to retinal cells. The antioxidant effects of anthocyanins theoretically could quench the singlet oxygen, which is released during chromovitrectomy [5–7]. The antioxidant property of the acai dye, associated with its high affinity for the internal limiting membrane (first observed in cadaveric eyes by our research team), became the basis for the development of a novel dye [8].

In 2017, our research group found that the 10% and 25% concentrations of the acai dye were safe when used in a rabbit model [9]. In this study, rabbits were injected intravitreously with the acai dye in three concentrations: 10%, 25%, and 35%. Control eyes received balanced salt solution. Functional evaluations were performed using electroretinography while morphologic examinations were performed by fundus imaging, fluorescein angiography, optical coherence tomography, light microscopy, and transmission electron microscopy. The 10% and 25% dye concentrations from the acai fruit did not cause significant functional or morphological toxicity. However, the 35% concentration showed evidence of morphologic and functional abnormalities suggestive of temporary toxic effects at 24 h follow-up.

Hence, the highest concentration of the acai fruit that was safe and effective in the rabbit model was 25%. This concentration was used for the sequence of a phase I/II clinical trial in 25 humans (unpublished data).

The objectives of the current study were to classify and quantify the anthocyanins of the acai fruit dye in the concentrations of 10%, 25%, and 35%. This information will become the basis for further understanding the antioxidant behavior of the acai dye.

## 2. Materials and Methods

### 2.1. Sample Preparation

The commercial acai extract was divided into flasks of 100, 250, and 350 mg of lyophilized anthocyanin powder to produce the concentrations of 10%, 25%, and 35%, respectively.

Ophthalmos SA (São Paulo, Brazil) developed the lyophilization process for the final dye preparation that was comprised mainly of anthocyanins. This process was performed in a sterile environment, stabilized at −50°C for 24 hours, and diluted in 1 ml of polyvinyl alcohol in the 10%, 25%, and 35% concentrations. The pH of the natural extract was acidic [10]. Hence, both the pH and osmolarity (pH 7.00; 300 mOsm) were controlled and established using one milliliter of phosphate-buffered solution (PBS). These concentrations also reflect the most suitable values obtained in preliminary studies in which factors such as staining capacity, pH, osmolarity, solubility, and stability were considered.

The lyophilized dye solutions were stored in depyrogenized amber flasks based on our previous results that anthocyanin molecules are highly unstable and light exposure might modify the staining capacity of the dye [9]. The anthocyanins were then analytically characterized by mass spectrometry.

### 2.2. Mass Spectrometry

Twenty-five-milligram samples of the raw materials and final products were subjected to mass spectrometry to compare the specific molecules in lower to higher quantities. This analysis was based on a spectral data bank. SGB Chemical Consultant Ltda. (São Paulo, Brazil), which was blinded to the dye components, analyzed the three lyophilized samples.

### 2.3. High-Performance Liquid Chromatography (HPLC)

Three microliters of dimethyl sulfoxide (DMSO) (Vetec Ventiltechnik GmbH) and 7.0 mL of the solution from the mobile phase were added to the flasks with the dye concentrations. These were then put in an ultrasonic bath, and the extraction process continued for 10 minutes. The flasks were removed from the bath, and the solutions were filtered into 1.5 mL vials containing a filter 0.22 micrometer in diameter. Finally, 20 *µ*L was added to the HPLC stabilized system.

The analysis was performed using the 1260 Infinity II liquid chromatography system and a diode array detector (Agilent Technologies, Santa Clara, CA). The specific methodology used a 5 *µ*m 150 × 4.6 mm Agilent Eclipse Column XDB C18 (Agilent Technologies). The mobile phase with an aqueous solution of 0.1% trifluoroacetic acid was referred to as phase A and acetonitrile HPLC was referred to as phase B.

The reagents used water (produced with purified water from a permutation technique), trifluoroacetic acid (Vetec Ventiltechnik GmbH, Speyer-am-Rhein, Germany), DMSO, and acetonitrile HPLC (Sigma-Aldrich, St. Louis, MO). This analysis used the gradients listed in Table 2, thus enabling quantification of all anthocyanins in the same chromatogram.

For the anthocyanin quantification using HPLC, it was first necessary to obtain the anthocyanin profiles using mass spectrometry. This information was used to determine the analytical standards of the anthocyanins which were then obtained from ChromaDex Inc. (Irvine, CA).

Five 25-milligram samples of the same major isolated anthocyanin molecules were used as comparative patterns from higher to lower quantities: cyanidin-3-O-glucoside, homoorientin, orientin, taxifolin, and isovitexin. The same amount (2.0 ± 0.20 mg) of the anthocyanin chemical standards was weighed in separate 10 mL volumetric flasks of 10 ml each. Subsequently, 3 mL of DMSO was added to each flask and subjected to high-frequency ultrasound for 5 minutes until the mobile phase. The patterns were mixed and diluted with the mobile phase.

The anthocyanin concentrations were quantified by HPLC using an electrochemical detector. The 350-nanometer wavelength was identified by the detector showing 1.00 mL/min flow by maintaining the column with the aid of a 40°C oven. The five molecules were used in the mobile phase of the solution in 10 ml/vial containing water and 30% DMSO without being retained in the filter. Such data were used to obtain the anthocyanin concentration for each concentration of the dye.

## 3. Results

### 3.1. pH and Osmolarity

Correction of pH and osmolarity and the lyophilization process were performed successfully.

### 3.2. Mass Spectrometry

**Figure 2:**
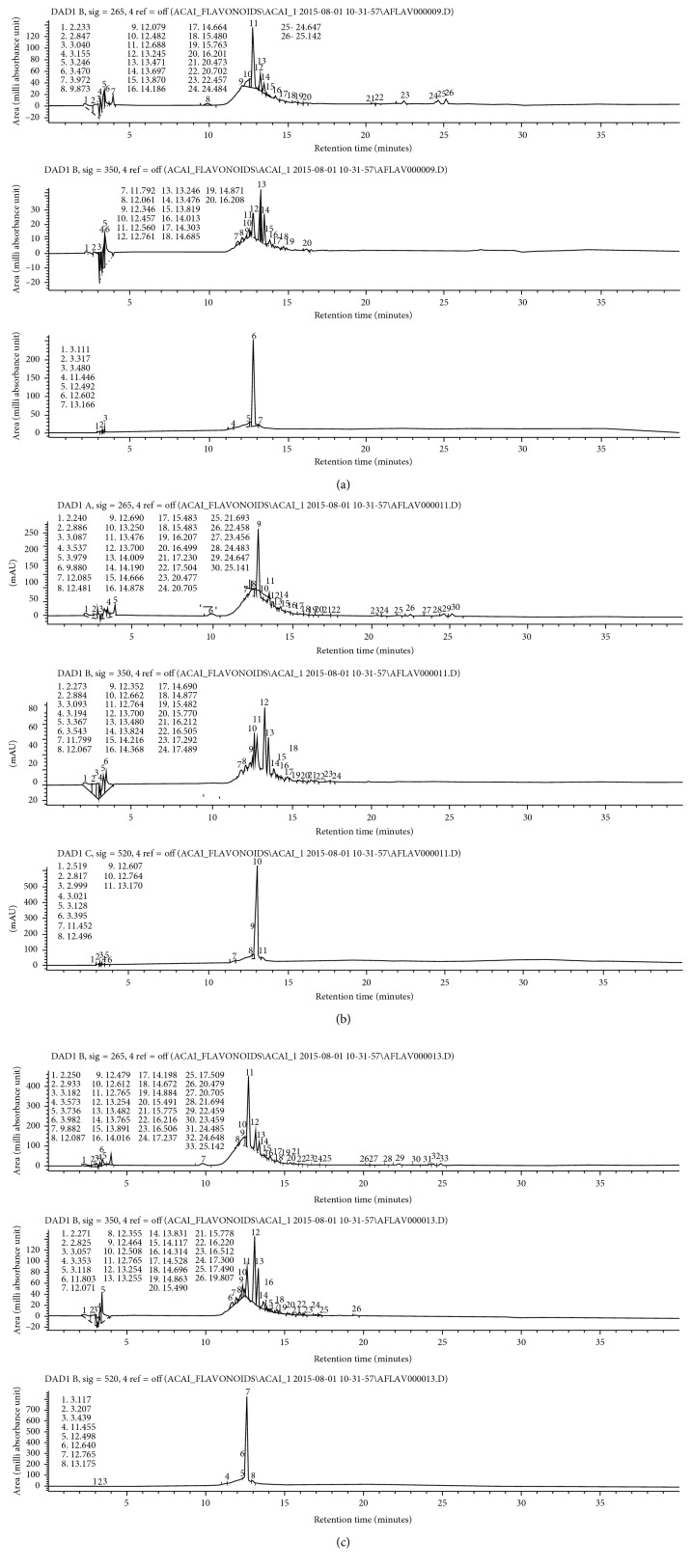
(a) A mass spectrometry graph of the 10% concentration of the vital dye extracted from the acai fruit. (b) A mass spectrometry graph of the 25% concentration of the vital dye extracted from the acai fruit. (c) A mass spectrometry graph of the 35% concentration of the vital dye extracted from the acai fruit.

This analysis showed the presence of five anthocyanins in the vital dye of the acai fruit from lower to higher quantities: taxifolin, orientin, isovitexin, homoorientin, and cyanidin-3-*O*-glucoside. Mass spectrometry analysis of the 10%, 25%, and 35% concentrations of the vital dye are shown in Figure 2.

### 3.3. HPLC

**Figure 3:**
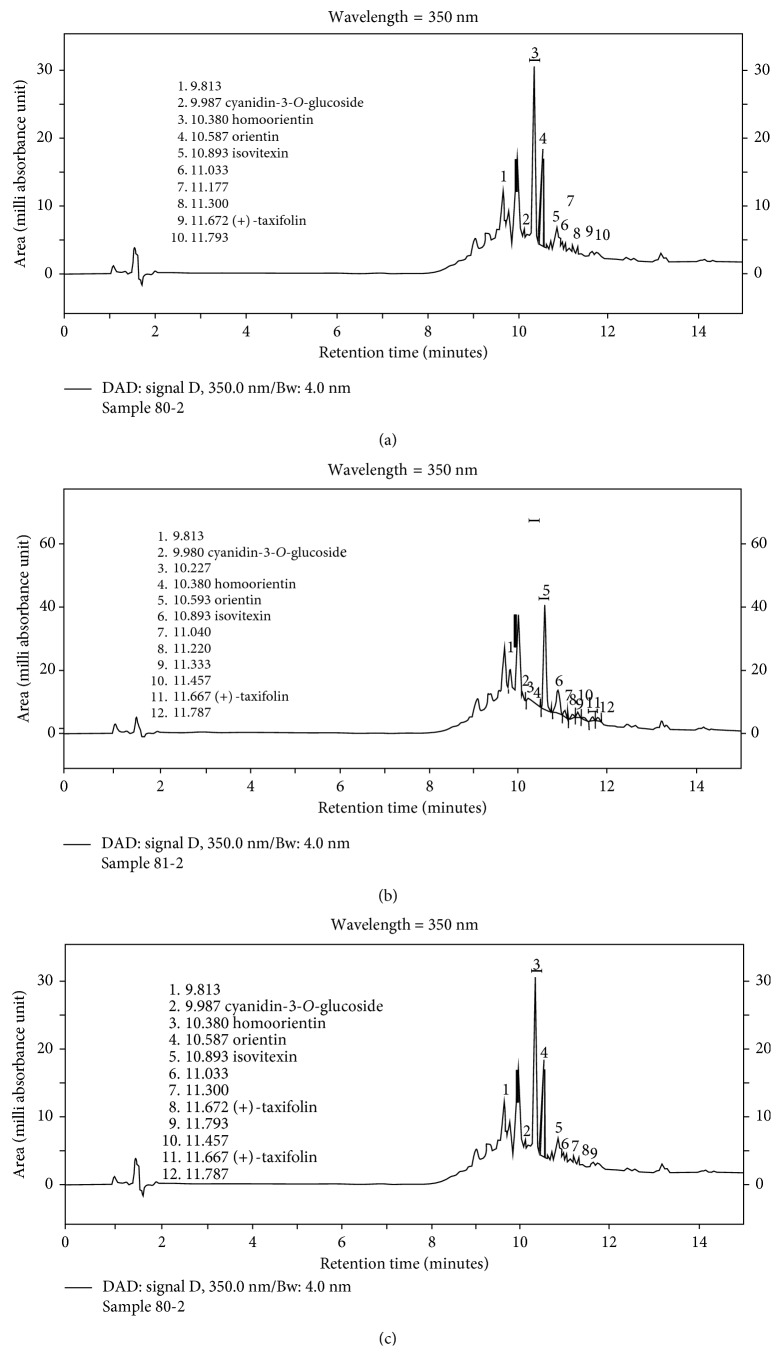
(a) A chromatograph of the 80-2 vital dye extracted from the acai fruit in a 10% concentration (equivalent to 100 mg of a lyophilized sample extracted from the acai pulp diluted in 1 ml PBS (pH 7.00; 300 mOsm). (b) A chromatograph of 81-2 vital dye extracted from the acai fruit in a 25% concentration (equivalent to 250 mg of a lyophilized sample extracted from the acai pulp diluted in 1 ml PBS (pH 7.00; 300 mOsm). (c) A chromatograph of 82-2 vital dye extracted from the acai fruit in a 35% concentration (equivalent to 350 mg of lyophilized extracted from the acai pulp diluted in 1 ml PBS (pH 7.00; 300 mOsm)).

This analysis quantified the five major anthocyanins in the vital dye of the acai fruit in the three tested concentrations based on the retention time by area shown in the chromatographs (Figures 3(a)–3(c)). Results showed that independent of the concentration of the acai dye, the anthocyanin concentration was similar (Table 3).

**Figure 4:**
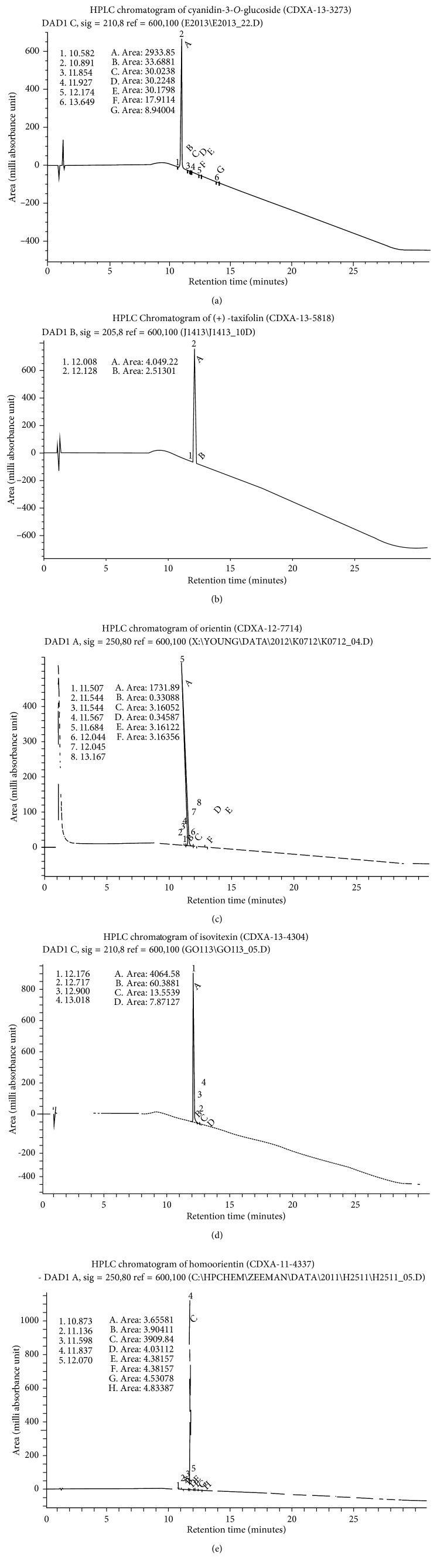
(a) A chromatograph of isolated anthocyanin molecules of cyanidin-3-O-glucoside 25 mg. (b) A chromatograph of isolated anthocyanin molecules of taxifolin 25 mg. (c) A chromatograph of isolated anthocyanin molecules of orientin 25 mg. (d) A chromatograph of isolated anthocyanin molecules of isovitexin 25 mg. (e) A chromatograph of isolated anthocyanin molecules of homoorientin 25 mg.

Chromatography results were compared with those of the isolated patterns, which facilitated confirmation of the presence of the anthocyanin molecules through separation of the flavonoids (Figures 4(a)–4(e)).

## 4. Discussion

The acai fruit is a multistemmed palm widely distributed in the northern South America. The fruit varies a great deal in the appearance of the plant, flowers, and fruiting products, which can be affected by genetic and environmental components. Other factors can affect the anthocyanin composition of fruits and their pulp, such as the harvest, type of soil, water quality, and spatial localization [11].

The current study aimed to characterize the physical-chemical composition of the anthocyanins present in the acai extract used in this research. The current data will be used to produce a standardized acai dye for use in chromovitrectomy that was first tested in cadaveric eyes [8] and rabbits [9] and now will be used in a phase I/II clinical trial in humans (unpublished data).

Mass spectrometry and HPLC identified and quantified the presence of five anthocyanins in the vital dye of the acai fruit: cyanidin-3-*O*-glucoside, homoorientin, orientin, isovitexin, and taxifolin. Data showed that cyanidin-3-*O*-glucoside is the major anthocyanin. These findings agreed with those of previous studies that also found that cyanidin-3-*O*-glucoside was the major anthocyanin found in the acai extracts [12, 13]. Results also showed that independent of the concentration of the acai dye, the anthocyanin concentration was similar (Table 3), and this result is probably because we employed the same prime material.

We hypothesized previously [8] that the anthocyanins of the acai fruit might have low potential for causing retinal and RPE toxicity in humans. Spectrophotometry analysis of different dye concentrations from the acai fruit had a peak light absorption around 1,000 nm; it is a fact that the lower the wavelength of the light source, the higher the energy emission and consequently the more toxic the light source is to the retina [7]. The high absorption peak of light from the acai solution suggests that this dye might have a safer toxicity profile regarding light absorption than other currently available dyes. A study that evaluated the absorbance spectra of nine vital dyes for chromovitrectomy found that most had two absorbance peaks, the first below 400 nm and the other in the visible light ranging from 400 to 700 nm [6].

In conclusion, the pharmacologic correction of the pH to 7.00 and osmolarity to 300 mOsm and the lyophilization process were successfully performed. The five major anthocyanins are present in the vital dye extracted from the acai, the major one being cyanidin-3-O-glucoside. Independent of the concentration of the acai dye, the anthocyanin concentration was similar. These standardized chemical characteristics of this new dye may allow its use during chromovitrectomy in humans. Further basic and clinical studies are needed to fully understand the role of acai dye anthocyanins and their antioxidant capacity.

## Figures and Tables

**Table 1:** Reference of nutritional values from 100 grams of acai fruit (*E. oleracea*) pulp on a diet of 2,500 kcal/day.

Protein	13 g
Iron	26 g
Fibers	34 g
Phosphorus	227 g
Sodium	56.4 g
Vitamin C	17 mg
Potassium	932 mg
Vitamin E	45 mg
Calcium	286 mg
Lipids	17 mg
Magnesium	174 mg
Glycides	36 mg
Calorie value	349 kcal

Source: Federal University of Para State, Brazil.

**Table 2:** Gradient used for liquid chromatography analysis of five major anthocyanins.

Time	Phase A (%)	Phase B (%)	Flow (mL/minute)
0.0	95	5	1.00
5.0	95	5	1.00
20.0	5	95	1.00
25.0	95	5	1.00
27.0	95	5	1.00
35.0	95	5	1.00

**Table 3:** Quantification of the anthocyanins based on the retention time by area of HPLC results at the three concentrations.

Anthocyanins	10% concentration	25% concentration	35% concentration
Cyanidin-3-*O*-glucoside			
Retention time (minutes)	9.987	9.980	9.973
Area (milli absorbance unit)	23.909	24.410	23.886

Homoorientin			
Retention time (minutes)	10.380	10.380	10.380
Area (milli absorbance unit)	38.511	36.950	40.106

Orientin			
Retention time (minutes)	10.587	10.593	10.580
Area (milli absorbance unit)	22.945	22.784	23.988

Taxifolin			
Retention time (minutes)	11.673	11.667	11.667
Area (milli absorbance unit)	0.966	0.894	0.998

Isovitexin			
Retention time	10.893	10.893	10.880
Area (milli absorbance unit)	6.755	6.737	6.194

## Data Availability

The data used to support the findings of this study are available from the corresponding author upon request.
